# Detection of Site-Specific Blood Flow Variation in Humans during Running by a Wearable Laser Doppler Flowmeter

**DOI:** 10.3390/s151025507

**Published:** 2015-10-05

**Authors:** Wataru Iwasaki, Hirofumi Nogami, Satoshi Takeuchi, Masutaka Furue, Eiji Higurashi, Renshi Sawada

**Affiliations:** 1 Advanced Manufacturing Research Institute, National Institute of Advanced Industrial Science and Technology, 807-1 Shuku-machi, Tosu, Saga 841-0052, Japan; 2 Department of Mechanical Engineering, Faculty of Engineering, Kyushu University, 744 Motooka, Nishi-ku, Fukuoka 819-0395, Japan; E-Mails: nogami@mech.kyushu-u.ac.jp (H.N.); sawada@mech.kyushu-u.ac.jp (R.S.); 3 Department of Dermatology, Graduate School of Medical Sciences, Kyushu University, 3-1-1 Maidashi, Higashi-ku, Fukuoka 819-8582, Japan; E-Mails: takeuchs@dermatol.med.kyushu-u.ac.jp (S.T.); furue@dermatol.med.kyushu-u.ac.jp (M.F.); 4 Department of Dermatology, Federation of National Public Service Personnel Mutual Aid Associations, Hamanomachi Hospital, 3-3-1 Nagahama, Chuo-ku, Fukuoka 810-8539, Japan; 5 Department of Precision Engineering, School of Engineering, The University of Tokyo, 7-3-1 Hongo, Bunkyo-ku, Tokyo 113-8656, Japan; E-Mail: eiji@su.t.u-tokyo.ac.jp

**Keywords:** wearable sensor, blood flow, exercise, laser Doppler, MEMS

## Abstract

Wearable wireless physiological sensors are helpful for monitoring and maintaining human health. Blood flow contains abundant physiological information but it is hard to measure blood flow during exercise using conventional blood flowmeters because of their size, weight, and use of optic fibers. To resolve these disadvantages, we previously developed a micro integrated laser Doppler blood flowmeter using microelectromechanical systems technology. This micro blood flowmeter is wearable and capable of stable measurement signals even during movement. Therefore, we attempted to measure skin blood flow at the forehead, fingertip, and earlobe of seven young men while running as a pilot experiment to extend the utility of the micro blood flowmeter. We measured blood flow in each subject at velocities of 6, 8, and 10 km/h. We succeeded in obtaining stable measurements of blood flow, with few motion artifacts, using the micro blood flowmeter, and the pulse wave signal and motion artifacts were clearly separated by conducting frequency analysis. Furthermore, the results showed that the extent of the changes in blood flow depended on the intensity of exercise as well as previous work with an ergometer. Thus, we demonstrated the capability of this wearable blood flow sensor for measurement during exercise.

## 1. Introduction

Vital monitoring of human health and activity using wearable sensors will be a key technology in the ubiquitous sensor network society for years to come. Recently, various noninvasive wearable sensors have been developed and applied to vital monitoring in various situations [1,2,3,4,5]. Many researchers have studied physiological effects during exercise using laser Doppler blood flowmeters, which are capable of noninvasive measurement of skin blood flow [6,7,8]. Although single point laser Doppler flowmetry (LDF) has less reproducibility than laser speckle contrast imaging (LSCI), LDF takes advantage of the ease of performing frequency analysis because it has a higher sampling frequency than LSCI [9,10,11]. A variety of information can be obtained from blood flow signals by conducting frequency analysis. Oscillations with frequencies of around 1.0, 0.3, 0.1, and 0.04 Hz represent heartbeat, respiration, vasomotion, and neurogenic activity, respectively [12]. Furthermore, the frequency interval between 0.021 and 0.052 Hz is of neurogenic origin [12,13,14,15]. In addition, we can obtain different blood flow signals between hairy and glabrous skin because of differences in the presence of arteriovenous anastomoses (AVA). This difference explains the physiological distinction between hairy and glabrous skin, the latter being found on the palms, soles of the feet, and the lips. Both sympathetic vasoconstrictor and sympathetic vasodilator nerves exist in hairy skin, but only sympathetic constrictor nerves are found in glabrous skin [16]. There are abundant AVA in glabrous skin at the fingertips, toes, and lips and the large amount of blood that can flow through the AVA causes large changes in skin blood flow, called a reflex wave [17]. Reflex waves appear in palmoplantar areas and are temporary and markedly decreased when, for example, the subject takes a deep breath or engages in physical activity [13]. In addition, it is possible to obtain sympathetic nervous activity from heart rate variability in blood flow [18].

When measuring blood flow during exercise, conventional blood flowmeters face several challenges, including large physical dimensions and high power consumption or considerable noise caused by movement of attached optic fibers [19,20,21]. Therefore, many different blood flowmeters have been used, primarily in experiments involving subjects on stationary bicycles rather than running, because the measuring points on the subject move less in the former situation.

To overcome these disadvantages, a laser Doppler blood flowmeter that eliminates the need for optic fibers was developed [22]. Combining the optic fiber-less approach with microelectromechanical systems (MEMS) technology, we developed a micro integrated laser Doppler blood flowmeter (a MEMS blood flow sensor) that is unaffected by movement because it does not use optic fibers [23,24,25]. We successfully used the MEMS blood flow sensor to evaluate dehydration, alcohol consumption, and systemic sclerosis [26,27,28]. Further, this compact wearable blood flow sensor can be applied to chicken health monitoring [29].

In this study, we attempted to expand the utility of the MEMS blood flow sensor by measuring more vigorous activities, such as running. We therefore conducted blood flow measurements during treadmill running for 30 min periods at various speeds (6, 8, and 10 km/h) as a pilot experiment. Furthermore, we compared the blood flow signals obtained from three different sites that differed in the existence of AVA, which greatly affects skin blood flow, and identified measurement sites that are suitable for monitoring blood flow during running [17].

## 2. Experimental Section

### 2.1. Subjects

The research work was approved by the research ethics committee of Kyushu University. Subjects were fully informed of the experimental details and any potential risks before their participation in the study. Seven healthy young men (mean age 23.4 years, range 22–26 years; mean body mass index 22.9 kg/m^2^, range 20.4–29.5 kg/m^2^) participated in this study. All subjects participated in sports activities less than once per week. They had breakfast more than 2 h before running and waited more than 30 min in the experimental room to become familiar with the environment and reach a relaxed state with regard to blood flow and heart rate.

### 2.2. MEMS Blood Flow Sensor

**Figure 1:**
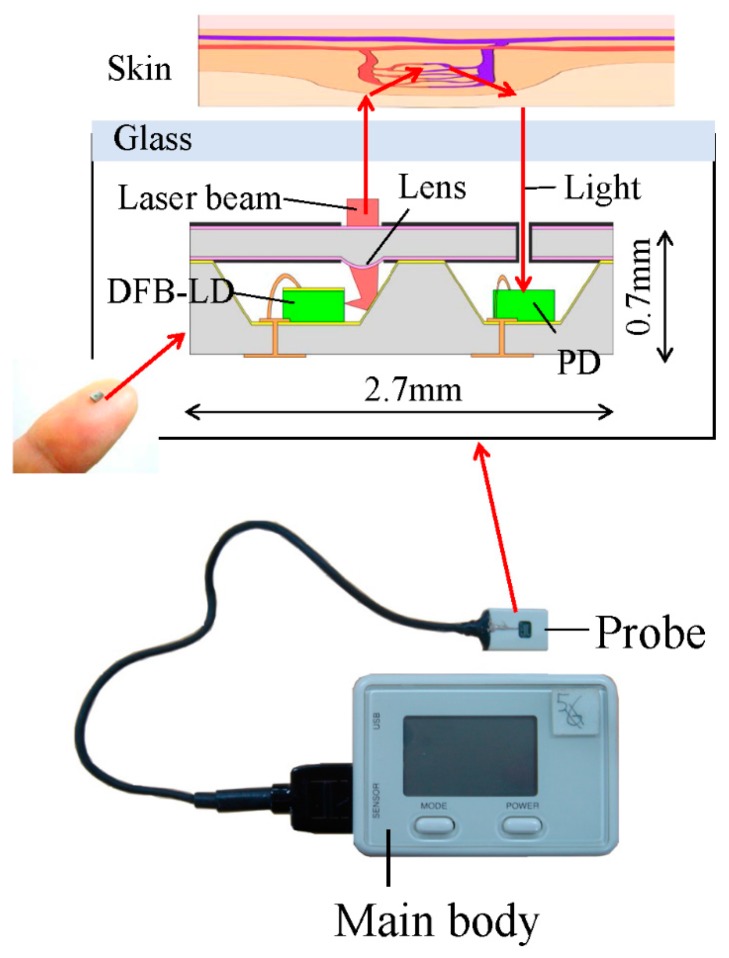
MEMS blood flow sensor. An image of the MEMS blood flow sensor (bottom) and schematic of the optical MEMS chip. The optical MEMS chip is located within the probe of the MEMS blood flow sensor. Silicon cavities are sealed with an upper silicon plate comprising a micro lens.

Figure 1 illustrates the MEMS blood flow sensor and its measurement principle [24]. The MEMS blood flow sensor consisted of a main body and a probe, with the main body composed of a Bluetooth wireless node, digital signal processor, display, and battery. The size of the main body was 60 × 40 × 25 mm and it weighed 50 g. The probe of the MEMS blood flow sensor, of dimensions 5.6 × 12 × 17.5 mm and weight 3 g, consisted of an optical MEMS chip and operational amplifier. The optical MEMS chip (2 × 2.7 × 0.9 mm) was fabricated with MEMS technology [24]. First, a silicon cavity with a (111) facet was fabricated by anisotropic wet etching with alkaline solution and then a through-silicon via (TSV) connecting the bottom surface of the silicon cavity and the underside of the silicon wafer was fabricated by deep reactive ion etching (RIE). The optical source was a distributed feedback laser diode (DFB-LD) with a wavelength of 1310 nm and photodiode (PD) bonded within the silicon cavities and sealed with an upper silicon plate. These DFB-LD and PD parts were electrically connected to the outside of the sealed silicon cavity by wire bonding and TSV. A silicon microlens 400 µm in diameter and 42 µm in height, for which the diameter was designed to be larger than the diameter of the laser beam emitted from the DFB-LD, was fabricated earlier on the upper plate by the following process. A photoresist with a microlens shape was patterned by a lithography and reflowing process. The shape was transferred onto the silicon wafer by RIE with SF_6_ and O_2_, yielding a silicon microlens. Finally, a 230 nm-thick SiO_2_ layer was deposited by sputtering as an anti-reflective coating to reduce surface reflection loss.

During operation, the laser beam emitted from the DFB-LD is reflected from the side walls of the silicon cavity, then irradiated onto the skin after focusing through the microlens of the upper silicon plate. The irradiated light penetrates the skin to a certain depth and is scattered from the skin, blood vessels, or red blood cells. The frequency of light scattered from the red blood cells is altered slightly by the Doppler effect, while light scattered from static tissue such as skin and blood vessels remains unchanged. The Doppler-shifted and non-shifted light signals interfere on the PD, and variations in light intensity caused by this interference are detected by the PD at a 40 kHz sampling rate. We can thereby obtain the blood flow rate as a proportion of the average velocity and concentration of red blood cells in the capillary from the optical signal using the following equation:

(1) $$Q=\frac{\int_{20}^{20k}fP(f)df}{I^{2}}$$

where *Q* is the blood flow rate, *f* is frequency, *P*(*f*) is the power spectrum of the optical signal, and *I* is the mean light intensity measured at the PD. The optical signal detected at the PD is transformed with a fast Fourier transformation (FFT) [30]. The first-order moment is calculated by integrating the frequency-weighted optical signal spectrum over the range of 20 Hz to 20 kHz. The first-order moment is divided by the square of mean light intensity measured at the PD. The blood flow rate is then wirelessly transferred to a personal computer at a 50 Hz sampling rate. Because the MEMS blood flow sensor does not use optic fibers, the motion artefacts are greatly reduced. Elimination of the optic fiber also contributes to a decrease in the coupling loss of the laser beam, therefore lowering the power consumption to 560 mW, which is about one-twentieth of that of a conventional blood flowmeter. The sensor thus runs for about 2 h on a single charge.

**Figure 2:**
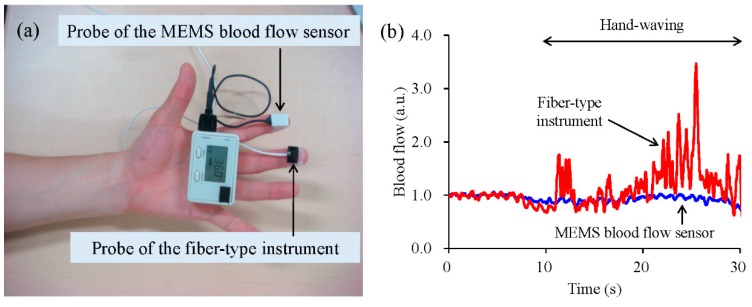
Comparison of blood flow signals between the MEMS blood flow sensor and a conventional optic fiber-type instrument [25]. A photograph of the probes from the MEMS blood flow sensor and an optic fiber-type instrument attached at the second and third fingertips, respectively (**a**); The blood flow signal patterns during hand waving are shown for each device (**b**). Hand waving was begun 10 s after the start of measurement.

Figure 2 shows the blood flow signals at the fingertip of a waving hand measured with the MEMS blood flow sensor and a conventional fiber-type instrument [25]. The probes of the MEMS blood flow sensor and the conventional fiber-type instrument were attached to adjacent fingertips (Figure 2a). Both signals were normalized by indicating the ratio to the blood flow at time zero to eliminate differences caused by differences in the optical features of the MEMS blood flow sensor and fiber-type instruments. The forearm was waved from the 10 s mark and it is apparent that the signal from the fiber-type instrument was greatly affected, while in contrast, the signal of our MEMS blood flow sensor was stable.

### 2.3. Experimental Designs

**Figure 3:**
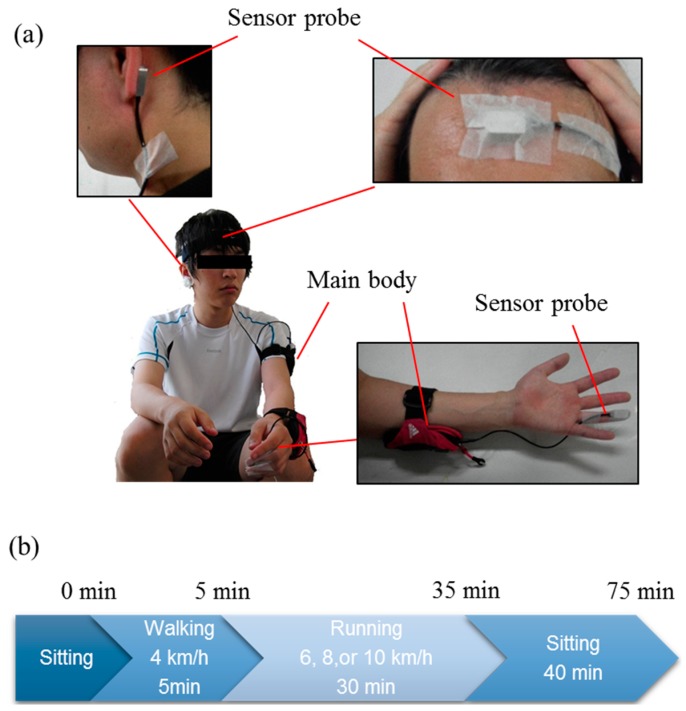
Actual pictures of running load experiment. Photographs of the attached MEMS blood flow sensors (**a**); and the experimental protocol (**b**). The MEMS blood flow sensor probes were attached using double-sided tape and surgical tape on the subject’s earlobe and the fourth fingertip or using a headband on the forehead. The main bodies of the MEMS blood flow sensor were put in the pocket-band on the left arm.

Participants ran on Tempo T931 treadmills (Johnson Health Tech. Co., Ltd., Taiwan) at controlled room temperature (mean 26.5 °C, range 24.0–28.4 °C). MEMS blood flow sensors were attached with double-sided tape and medical tape to the tip of the left ring finger, the left earlobe, and the forehead using a headband, as shown in Figure 3a. In addition to blood flow, we measured body temperature and body weight. Body temperature was measured with an MC-510 ear thermometer (Omron Healthcare Co., Ltd., Kyoto, Japan) which can measure body temperature within 1 s, making it usable even while running.

We measured blood flow at three different running velocities (6, 8, and 10 km/h) for 30 min each after walking for 5 min at 4 km/h as a warm-up. Although 6 km/h is actually a brisk walking speed, we had the subjects run, not walk. For the subjects in this experiment, 10 km/h was a sufficiently high speed because they did not regularly engage in sports activities. The metabolic equivalents (METS) of running at 6, 8, and 10 km/h are approximately 5, 8, and 10, respectively [31,32]. The intensity level of exercise for these METS values is assessed as “moderate”, “very heavy”, and “unduly heavy” for men who do not exercise regularly [33]. Subjects ran each velocity trial on different days, with intervals of more than three days between sessions to counteract fatigue from the previous experiment.

Figure 3c shows the experimental protocol. At the beginning of each session, MEMS blood flow sensors were attached to the subject, followed by measurement of resting blood flow and body temperature while the subject was sitting. This was followed by the subject walking on the treadmill at 4 km/h for 5 min. The body temperature was measured 4 min after the onset of walking. The subject ran for 30 min at a constant velocity after the warm-up phase and the body temperature was measured 5, 10, 15, 20, 25, and 29 min after running commenced. We monitored blood flow while subjects sat for 40 min after running to monitor the recovery of blood flow. Body temperature was also measured immediately, 20, and 40 min after completion of running. If subjects complained of exhaustion while running, we stopped the running session but continued to monitor blood flow for 40 min.

### 2.4. Analysis

We conducted short-time Fourier transform (STFT) analyses on blood flow signal data during running to observe the changes in heart rate and basic wave signals. The wavelet transform (WT) is commonly used in frequency analysis of blood flow signals to investigate the time-spectrum distribution in the low frequency region [12]. However, WT has poor frequency resolution in the high-frequency area. Because we were interested in observing the spectrum peak derived from the heart rate, which has a high frequency under running conditions, we did not perform WT, but STFT. The frame length of the STFT was 40.96 s (2048 points), the overlap between successive data sets was 50% (1024 points), and the Hamming window was used. Band-pass filters from 1.0 to 2.0 Hz, 2.0 to 3.5 Hz, and 1.5 to 3.5 Hz were used to observe the heart rate signals before, during, and after running, respectively.

## 3. Results

### 3.1. Stability of Blood Flow Signals at Each Measurement Site

**Figure 4:**
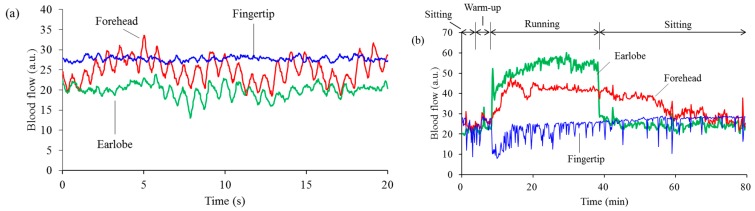
Differences in blood flow between measurement sites. Typical examples of basal blood flow in a sitting condition, before walking (**a**); and blood flow while the subject was running at 10 km/h (**b**) at the fingertip (blue), at the forehead (red), and at the earlobe (green).

The blood flow signals at each measurement site when a representative subject ran at 10 km/h are shown in Figure 4. Figure 4a shows basal blood flow measured in a sitting condition before walking. Because there are many capillaries at the fingertip, basal blood flow at the fingertip was the highest. Further, capillaries undergo little pulsatile motion, and there is only a minor cardiac wave in the blood flow at the fingertip. The data was sampled at 50 Hz, and the values displayed were averaged every 500 points, and plotted at a 10 s interval for clarity in Figure 4b. Blood flow at the fingertip decreased at the beginning of running and then gradually increased. There were many temporary decrements of blood flow at the fingertip, caused by reflex waves. Many reflex waves appeared during running because physical activity stimulated the parasympathetic nervous system. Sometimes, a reflex wave also appeared even in a sitting condition, caused by the subject taking a deep breath. Blood flow at the forehead gradually increased from the start of the run, maintained a stable signal throughout the test, and gradually decreased after the subject finished running. The blood flow at the earlobe suddenly and markedly increased upon commencing the run.

### 3.2. Separation of Heartbeat from Heart Rhythm while Running

**Figure 5:**
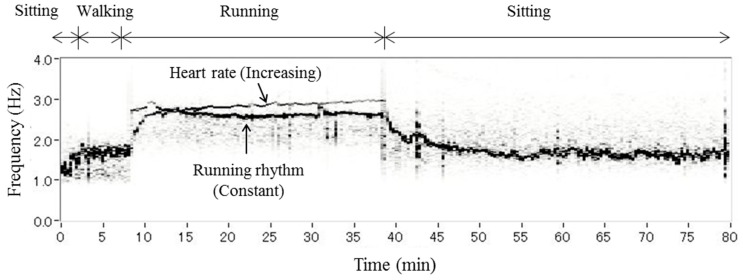
Frequency of blood flow while running. Typical example of the time-spectrum distribution of blood flow at the forehead while a subject ran at 10 km/h. The black color depth represents the intensity. These spectra were obtained by performing FFTs on blood flow signal data lasting for 40.96 s (2048 points). Band-pass filters from 1.0 to 2.0 Hz during the first sitting and walking period, from 2.0 to 3.5 Hz during running, and from 1.5 to 3.5 Hz during the second sitting period were applied.

Normally, we would see a single spectral peak at around 1 Hz when performing a FFT on blood flow, representing the heartbeat. However, we found several additional spectral peaks in the blood flow signal while running. Figure 5 shows the time-spectrum distribution of blood flow at the forehead of a subject while running at 10 km/h, obtained by STFT. Band-pass filters from 1.0 to 2.0 Hz during the initial sitting and walking period, from 2.0 to 3.5 Hz during running, and from 1.5 to 3.5 Hz during the second sitting period were applied. There was a single strong signal until the subject started running, followed by two strong spectra. One of the spectra had a constant frequency, while the other showed an increase in frequency. The latter represents the heart rate, while the former, constant peak represents the running rhythm. We have thus succeeded in separating the heart rate and motion artifact by performing STFT on blood flow and observing the changing frequencies of the spectral peaks. There was an apparent decrease in the heart rate spectra from 3 Hz to around 1.5 Hz after subjects finished the running session.

### 3.3. Differences among Running Speeds

**Figure 6:**
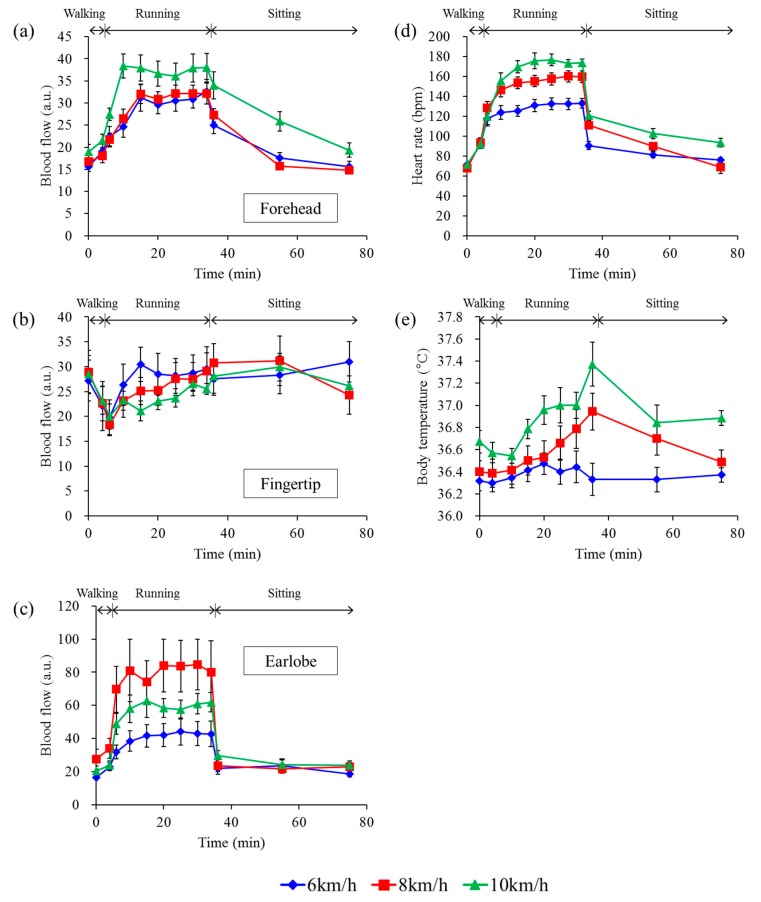
Changes in blood flow and other parameters at each running speed. Mean blood flow at the forehead (**a**); the fingertip (**b**); and the earlobe (**c**); heart rate (**d**); and body temperature (**e**). Error bars represent standard error. Seven subjects ran at 6 km/h (blue rhomboids), 8 km/h (red squares), and 10 km/h (green triangles) for 30 min. Two of the seven subjects gave up 15 and 20 min after running started at 10 km/h because of difficulty maintaining the pace. Therefore, seven data sets collected at each time point, except for 20 min at 10 km/h (*n* = 6) and from 25 min to 34 min at 10 km/h (*n* = 5).

Figure 6 shows the mean blood flows measured at the forehead (a), at the fingertip (b), and at the earlobe (c), heart rate (d), and body temperature (e) at each running velocity. We observed a change in mean blood flow of all subjects at each time point. Blood flow data over a period of 40.96 s (2048 points) were averaged in each subject, and the averaged blood flow of all subjects was further averaged. Each time point shows sitting before walking (0 min), walking (4 min), running just started (6 min), 5, 10, 15, 20, 25 min after running started (10, 15, 20, 25, 30 min), immediately before running finished (34 min), and 1, 20, and 40 min after running finished (36, 55, 75 min). Heart rate was obtained by FFT of the blood flow measured at the forehead. All subjects completed the 30 min run at 6 and 8 km/h, but during the 10 km/h session, two subjects retired 15 and 20 min after running started, respectively, while other subjects completed the session. Therefore, there were seven data sets collected at each time point, except for 20 min at 10 km/h (*n* = 6) and from 25 min to 34 min at 10 km/h (*n* = 5). The blood flow at the forehead gradually increased while running at each velocity, with the 10 km/h session yielding the highest flow. In contrast, the blood flow at the fingertip dropped suddenly, then gradually increased when running at each velocity, with the 10 km/h session yielding the slowest rate of increase. The mean heart rate increased while running at each velocity and the magnitude depended on the running speed. The mean heart rate at 10 km/h rose over 160 bpm within 10 min after running commenced. The mean body temperature rose to over 37.0 °C in the latter stages of the 10 km/h running session. The coefficient of variation (CV) in individual subjects at each measurement site was also calculated from the baseline blood flow measured before the walking session (time = 0 min). The individual CVs in blood flow at the forehead, fingertip, earlobe, and heart rate ranged from 4.0%–25.3%, 6.1%–27.2%, 5.0%–47.7%, and 3.6%–14.7%, respectively.

## 4. Discussion and Conclusions

The blood flow signals differed at the three measurement sites. The changes in blood flow at the forehead at the beginning and end of running were mild, but those at the earlobe were sudden and significant. Furthermore, the changes in blood flow at the earlobe did not seem to be linearly correlated with the degree of running speed. We believe that the sudden changes at the earlobe were caused by shear deformation of the skin. Running motion would easily cause shear deformation of the skin at the earlobe, because the skin is very soft and it is difficult to hold the probe steady in that area. The blood flow sensor may thus have detected this relative movement. In contrast, the blood flows at the forehead, where a robust positioning was made possible by medical tape and headbands, were only slightly affected by the running motion (Figure 4). However, blood flow measurements at the fingertip were also unaffected by motion artifacts. Motion artifacts cause increased apparent blood flow, while in contrast, blood flow at the fingertip initially decreased upon the start of motion, and subsequently increased. It is considered that differences between increasing and decreasing blood flow signals during running are caused by differences in physiological characteristics between hairy and glabrous skin. Both sympathetic vasoconstrictor and sympathetic vasodilator nerves exist in hairy skin but only sympathetic constrictor nerves exist in glabrous skin [16]. Usually, the entire skin blood flow is controlled by the function of the sympathetic vasoconstrictor nerves. When body temperature increases, blood flow at the hairy skin will increase only through the function of sympathetic vasodilator nerves for heat release [34]. Moreover, the abundant AVA in glabrous skin at the fingertips causes large drops in skin blood flow [17] and when a subject starts running, a large amount of blood flow is sent to the muscles [8]. Therefore, we conclude that the forehead is a suitable measurement site for blood flow while running or engaging in other activities with significant motions, because we can measure stable blood flow signals and motion artifacts are greatly reduced. In addition, the fingertip is also a candidate for a second measurement site because blood flow at the fingertip undergoes fewer motion artifacts and shows a different physiological response, which would be helpful in obtaining additional physiological information. In contrast, the earlobe is unsuitable as a measurement site because it is significantly affected by motion artifacts.

In conclusion, we attempted to observe blood flow during running using our MEMS blood flow sensor. We succeeded in achieving stable blood flow measurements on running subjects. The MEMS blood flow sensor also captured the noise in the signal caused by the running motion but we could readily distinguish between this noise and the pulse wave by conducting STFT on blood flow and observing an incremental frequency shift in the latter spectral peak. Moreover, we found that the extent of the changes in blood flow measured at 10 km/h, which is a sufficient speed for subjects who do not engage in regular exercise to become tired and quit running, was higher than that measured at other speeds as well as previous work with an ergometer [35]. These results demonstrate that the MEMS blood flow sensor has the potential for use as a new wearable device to monitor health-related information during exercise. Further, we compared blood flow signals measured at different sites on the body and showed that the forehead is suitable for measuring blood flow during running in contrast to the earlobe. These results are expected to be helpful in future physiological research work carried out with a blood flow meter during running.
